# Early lactate measurement is associated with better outcomes in septic patients with an elevated serum lactate level

**DOI:** 10.1186/s13054-019-2625-0

**Published:** 2019-11-11

**Authors:** Hui Chen, Chenyan Zhao, Yao Wei, Jun Jin

**Affiliations:** Department of Intensive Care Medicine, The First Affiliated Hospital of Soochow University, No. 899 Pinghai Road, Suzhou, 215000 Jiangsu China

**Keywords:** Lactate measurement, Remeasurement, 28-day mortality, Septic patients

## Abstract

**Background:**

The optimal timing of lactate measurement for septic patients in the intensive care unit (ICU) remains controversial, and whether initiating and repeating the lactate measurement earlier could make a difference for septic patients with an elevated lactate level remains unexplored.

**Methods:**

This was a retrospective observational study that included septic patients with an initial lactate level > 2.0 mmol/L after ICU admission, and all data were extracted from the Medical Information Mart for Intensive Care III (MIMIC-III) database. The main exposure of interest was the early lactate measurement, which was defined as an initial lactate level measurement within 1 h after ICU admission. The primary outcome was 28-day mortality.

**Results:**

A total of 2642 eligible subjects were enrolled, including 738 patients who had initial lactate measurements completed within 1 h (EL group) and 1904 patients who had initial lactate measurements completed more than 1 h after ICU admission (LL group). A significant beneficial effect of early lactate measurement in terms of 28-day mortality was observed: the adjusted odds ratio (OR) was 0.69 (95% CI 0.55–0.87; *p* = 0.001), and the mediation effect of the time to initial vasopressor administration was significant (average causal mediation effect (ACME) − 0.018; 95% CI − 0.005 approximately to − 0.036; *p* < 0.001). A strong relationship between delayed initial lactate measurement and risk-adjusted 28-day mortality was noted (OR 1.04; 95% CI 1.02–1.05; *p* < 0.001). Each hour of delay in remeasuring the lactate level was associated with an increase in 28-day mortality in the EL group (OR 1.09; 95% CI 1.04–1.15; *p* < 0.001). Further analysis demonstrated that repeating the measurement 3 h after the initial lactate measurement led to a significant difference.

**Conclusions:**

Early lactate measurement is associated with a lower risk-adjusted 28-day mortality rate in septic patients with lactate levels > 2.0 mmol/L. A shorter time to the initial vasopressor administration may contribute to this relationship. Repeating the lactate measurement within 3 h after the initial measurement is appropriate for patients whose lactate levels were measured within 1 h of admission.

## Introduction

Sepsis is one of the leading causes of in-hospital death in the intensive care unit (ICU), with a short-term mortality rate ranging from 30 to 50% [1, 2]. Elevation as well as slow clearance of serum lactate levels has been reported to be associated with increased mortality in sepsis [3, 4]. As a product of anaerobic metabolism, lactate elevation can to some extent reflect poor tissue perfusion, though it can be affected by other factors [5]. Thus, monitoring lactate levels may help clinicians understand the tissue perfusion situation and identify unrecognized shock, enabling them to adjust treatment in a timely fashion.

Because randomized controlled trials have demonstrated a significant reduction in mortality with lactate-guided resuscitation [6, 7], lactate measurement has been introduced into the Surviving Sepsis Campaign (SSC) guidelines as an important component of the treatment bundles. The 2012 SSC bundle emphasized that an initial lactate level must be measured between 6 h before and 3 h after sepsis onset, followed by a repeated measurement within 6 h of sepsis presentation if the initial value is elevated [8]. Levy et al. updated the SSC bundle to indicate that the initial lactate measurement should be performed within 1 h of onset and remeasured within 2–4 h to guide resuscitation if the initial lactate is > 2.0 mmol/L [9]. However, there are limited studies focused on the timing of lactate measurement and remeasurement and its effect on outcomes. One study by Han et al. [10] demonstrated that patients with severe sepsis or septic shock and initial lactate levels > 2.0 mmol/L had a 2% increase in the odds of death with each hour delay in lactate measurement. According to the analysis of 96 studies, Vincent et al. concluded that lactate measurement every 1–2 h is probably sufficient in most acute conditions, including sepsis [11]*.* However, the optimal timing of the initial lactate measurement for septic patients remains controversial, and whether earlier initial and repeated lactate measurements could make a difference for septic patients with a higher lactate level remains unexplored.

Therefore, we designed this study to examine the relationship between early lactate measurement (within 1 h after ICU admission) and the outcomes of septic patients with an elevated serum lactate level (> 2.0 mmol/L), as well as to characterize the association of delays in initial lactate measurement and remeasurement with 28-day mortality to better understand the benefit of lactate monitoring in sepsis management.

## Methods

### Study design

This was a retrospective observational study in which data were extracted from an online international database, the Medical Information Mart for Intensive Care III (MIMIC-III) [12]. The MIMIC-III is a large, single-center database that comprises information related to patients admitted to critical care units at a large tertiary care hospital located in Boston. The database contains 53,423 distinct hospital admissions for adult patients (age 16 years or above) admitted to critical care units between 2001 and 2012. It is possible to access this database by passing an examination and obtaining the certification. One author (HC) obtained access and was responsible for the data extraction (certification number 27252652).

### Selection of participants

Septic patients with an initial lactate level > 2.0 mmol/L after ICU admission were eligible for inclusion in our study. The diagnoses of sepsis were consistent with the third sepsis definition [13], that is, patients with documented or suspected infection and an acute change in total SOFA score ≥ 2 points. Infection was identified from the ICD-9 code in the MIMIC-III database. Patients who were younger than 18 years or spent fewer than 48 h in the ICU were excluded. For patients who were admitted to the ICU more than once, only the first ICU stay was included for analysis. Included patients were subsequently divided into two groups: the early lactate group (EL group, the initial lactate level was measured within 1 h after ICU admission) and the late lactate group (LL group, the initial lactate level was measured more than 1 h after ICU admission).

### Variable extraction

Data from the MIMIC-III database that were considered baseline characteristics within the first 24 h after ICU admission included the following: gender, weight, race, admission type, admission period, severity at admission measured by Sequential Organ Failure Assessment (SOFA) score, quick Sequential Organ Failure Assessment (qSOFA) score, Simplified Acute Physiology Score II (SAPS II) score, Overall Anxiety Severity and Impairment Scale (OASIS) score, Elixhauser comorbidity score, use of mechanical ventilation, use of renal replacement therapy (RRT), and administration of vasopressors. Initial lactate levels and vital signs, including mean arterial pressure (MAP), heart rate, temperature (°C), and respiratory rate, were also extracted. If a variable was recorded more than once in the first 24 h, we used the value related to the greatest severity of sepsis.

Comorbidities including congestive heart failure (CHF), atrial fibrillation (AFIB), chronic renal disease, liver disease, chronic obstructive pulmonary disease (COPD), stroke, and malignant tumor were identified on the basis of the recorded ICD-9 codes. The site of infection and blood culture data were also collected for analysis.

### Outcomes and therapeutic interventions

The primary exposure was the early lactate measurement, which was defined as an initial lactate level measured within 1 h after ICU admission. The primary outcome of the present study was 28-day mortality. Secondary outcomes included mechanical ventilation-free days and vasopressor-free days within 28 days after ICU admission, AKI stage, and the duration of ICU and hospital stays.

Therapeutic interventions in our study might have transmitted the effect of early lactate measurement to the primary outcome, including the time to initial vasopressor administration (hours), time to initial antibiotic therapy (hours), time to initial intravenous fluid (IVF) treatment (hours), and volume (L) of IVF administered within 6 h and 24 h of ICU admission.

The details of missing data are summarized in Additional file 1: Table S1. More than 20% of the missing data were removed from our analysis, and the remaining missing data were obtained with the multiple imputation method.

### Causal mediation analysis

Causal mediation analysis (CMA) [14] is a method for separating the total effect of a treatment into direct and indirect effects. The indirect effect on the outcome is mediated via a mediator. The analysis reports consist of the average causal mediation effect (ACME), average direct effect (ADE), and total effect. In our study, we used the early lactate measurement as the treatment and the time to initial IVF, time to initial antibiotic treatment, and time to initial vasopressor administration as mediator variables to explore whether the effect of early lactate measurement on the primary outcome is mediated by the mediator variables mentioned above.

### Statistical analysis

Continuous variables are expressed as the mean ± standard deviation or median (interquartile range), as appropriate. Categorical variables are shown as proportions. Student’s *t* test, analysis of variance, and the Mann-Whitney *U* test were used as appropriate. Categorical variables were compared using the *χ*^2^ test.

Multivariate modeling of the association between early lactate measurement and 28-day mortality was performed with logistic regression. Baseline variables that were considered clinically relevant or that showed a univariate relationship with the outcome (*p* < 0.10) were entered into a multivariate logistic regression model as covariates, and included age, gender, weight, admission type, admission period, severity scores, use of mechanical ventilation, use of RRT, use of vasopressors, comorbidities, site of infection, MAP, and initial lactate level. The variance inflation factor (VIF) method was used to examine multicollinearity, and VIF ≥ 5 suggested multicollinearity in our model. Subgroup analyses according to gender, admission period, comorbidities, vasopressor use, and site of infection were performed. We also investigated the relationship between a delay in initial lactate measurement and a delay in remeasurement in the EL group and 28-day mortality by multivariate logistic regression.

Propensity score matching (PSM) [15] was used to minimize the imbalance of covariates between the EL and LL groups. A multivariate logistic regression model was used to estimate the patient’s propensity scores for early lactate measurement. A 1:1 nearest neighbor matching was applied with a caliper width of 0.02 in our study. The standardized mean differences (SMDs) and *p* values were calculated to evaluate the effectiveness of the PSM. We then used the PSM model and the doubly robust model [16, 17]—the combination of the multivariate logistic regression model and PSM model—to further clarify the relationship between early lactate measurement and 28-day mortality. Outcomes and therapeutic interventions were generated from a matched cohort. CMA was employed to explore the association described above.

All statistical analyses were performed using the R package (version 3.6.0), and *p* < 0.05 was considered statistically significant.

## Results

### Demographic data and baseline characteristics

**Fig. 1:**
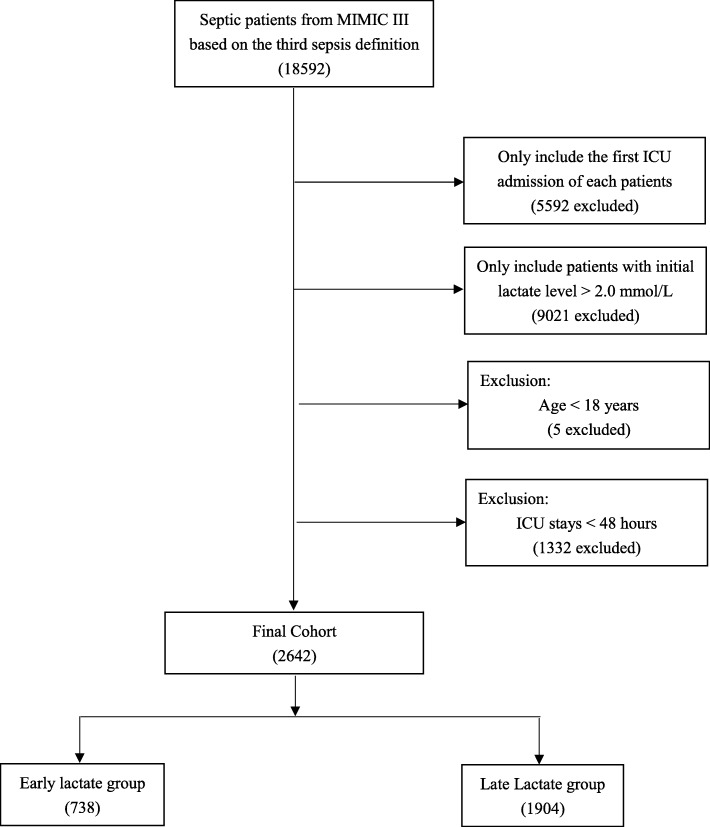
The flow diagram of study patients

A total of 2642 septic patients with elevated serum lactate levels were enrolled in our study cohort. The flow diagram of study patients is presented in Fig. 1. Of the study cohort, 738 patients (27.9%) had initial lactate measurements completed within 1 h and 1904 patients had measurements completed more than 1 h after ICU admission. The overall 28-day mortality rate was 26.8%. The characteristics of the EL and LL groups are summarized in Table 1. In general, patients in the EL group had a significantly higher SOFA sore (7.8 (3.6) vs. 7 (3.6); *p* = 0.002) and initial lactate level (3.6 (2.7–5.2) vs. 3.1 (2.5–4.4); *p* < 0.001) on admission. Within the first 24 h after ICU entry, the EL group was more likely to use mechanical ventilation (80.1% vs. 74.1%; *p* = 0.001) and vasopressors (62.3% vs. 57.7%; *p* = 0.034). Respiratory infection (46.6% vs. 38.9%; *p* < 0.001) was more common in the EL group, while more patients in the LL group had urinary infections (20.3% vs. 24.5%; *p* = 0.025) and infection at other sites (21.8% vs. 27%; *p* = 0.007). Additional demographic data compared between the groups are presented in Additional file 1: Table S2.

**Table 1:** Demographic data and comparisons between the early lactate group and the late lactate group before matching

Variables	EL group (*n* = 738)	LL group (*n* = 1904)	*p* value	SMD
Age (years)	67 (53–78)	68 (54–79)	0.266	0.051
Male, *n* (%)	424/738 (57.5)	1020/1904 (53.6)	0.079	0.078
Weight (kg)	83.1 (67.1–94)	79.9 (66–95)	0.836	0.032
Admission type, *n* (%)			0.328	0.064
Emergency	631/738 (85.5)	1666/1904 (87.5)		
Surgical elective	84/738 (11.4)	180/1904 (9.4)		
Surgical urgency	23/738 (3.1)	58/1904 (3)		
Admission period, *n* (%)			0.737	0.016
Before 2008	437/738 (59.2)	1112/1904 (58/4)		
2008–2012	301/738 (40.8)	792/1904 (41.6)		
Race, *n* (%)			0.873	0.048
White	523/738 (70.9)	1315/1904 (69)		
Asian	18/738 (2.4)	47/1904 (2.5)		
Black	56/738 (7.6)	149/1904 (7.8)		
Hispanic	24/738 (3.2)	71/1904 (3.7)		
Other	117/738 (15.8)	322/1904 (16.9)		
Severity of illness				
SOFA score	7.8 (3.6)	7 (3.6)	0.002	0.136
qSOFA score	2 (2–2)	2 (2–2)	0.669	0.030
SAPS II score	47.7 (15)	47.2 (14.6)	0.454	0.033
OASIS score	38.6 (8.27)	38.4 (8.43)	0.618	0.022
Elixhauser comorbidity score	11.4 (8.61)	11.5 (8.68)	0.949	0.003
Interventions, *n* (%)				
RRT use (1st 24 h)	40/738 (5.4)	111/1904 (5.8)	0.754	0.018
Mechanical ventilation use (1st 24h)	591/738 (80.1)	1410/1904 (74.1)	0.001	0.144
Vasopressor use (1st 24 h)	460/738 (62.3)	1099/1904 (57.7)	0.034	0.094
Comorbidities, *n* (%)				
CHF	239/738 (32.4)	634/1904 (33.3)	0.688	0.019
AFIB	296/738 (40.1)	823/1904 (43.2)	0.158	0.063
Chronic renal disease	111/738 (15)	365/1904 (15)	0.999	0.009
Liver disease	153/738 (20.7)	286/1904 (22.5)	0.342	0.001
COPD	139/738 (18.8)	429/1904 (19.2)	0.887	0.044
Stroke	15/738 (2)	51/1904 (2.7)	0.415	0.043
Malignancy	90/738 (12.2)	217/1904 (11.4)	0.612	0.025
Septic shock, *n* (%)	483/738 (65.4)	1167/1904 (61.3)	0.053	0.086
Site of infection, *n* (%)				
Respiratory	344/738 (46.6)	740/1904 (38.9)	< 0.001	0.157
Urinary	150/738 (20.3)	467/1904 (24.5)	0.025	0.101
Gastrointestinal	83/738 (11.2)	182/1904 (9.6)	0.221	0.055
Other*	161/738 (21.8)	515/1904 (27)	0.007	0.122
Positive blood cultures, *n* (%)	242/738 (32.8)	595/1904 (31.2)	0.473	0.033
Vital signs				
MAP (mmHg)	76.2 (9.6)	75.7 (10.2)	0.190	0.056
Heart rate (bpm)	94.8 (17)	93.5 (17.2)	0.084	0.075
Temperature (°C)	36.9 (0.8)	36.9 (0.8)	0.655	0.020
Respiratory rate (bpm)	20.3 (4.8)	20.3 (4.64)	0.988	0.001
Initial lactate level (mmol/L)	3.6 (2.7–5.2)	3.1 (2.5–4.4)	< 0.001	0.261

*SOFA* Sequential Organ Failure Assessment, *qSOFA* quick Sequential Organ Failure Assessment, *SAPS II* Simplified Acute Physiology Score II, *OASIS* Overall Anxiety Severity And Impairment Scale, *RRT* renal replacement therapy, *CHF* congestive heart failure, *AFIB* atrial fibrillation, *COPD* chronic obstructive pulmonary disease, *MAP* mean arterial pressure

*Other site of infection included central nervous system, skin, soft tissue, and unknown

### Associations between early lactate measurement and the primary outcome

Univariate and multivariate logistic regression analyses showed a significant beneficial effect of early lactate measurement in terms of the 28-day mortality, and the adjusted odds ratio (OR) was 0.69 (95% CI 0.55–0.87; *p* = 0.001) (Additional file 1: Table S3). After PSM, 701 patients in the EL group were matched to 701 patients in the LL group by a 1:1 matching algorithm (Additional file 1: Table S4, Figure S1, and Figure S2). When using PSM analysis and doubly robust analysis, the association remained significant (Table 2). Details of the double robust model are presented in Additional file 1: Table S5. We also constructed a sensitivity analysis to investigate patients with positive blood culture and drew the same conclusion (Additional file 1: Table S6).

**Table 2:** Association between early lactate measurements and 28-day mortality with different models

	Odds ratio	95% CI	*p* value
Model 1	0.69	0.55–0.87	0.001
Model 2	0.75	0.59–0.96	0.022
Model 3	0.70	0.53–0.93	0.013

Model 1, multivariate logistic regression model; Model 2, propensity score matching model; Model 3, doubly robust model with all covariates. *CI* confidence interval

**Fig. 2:**
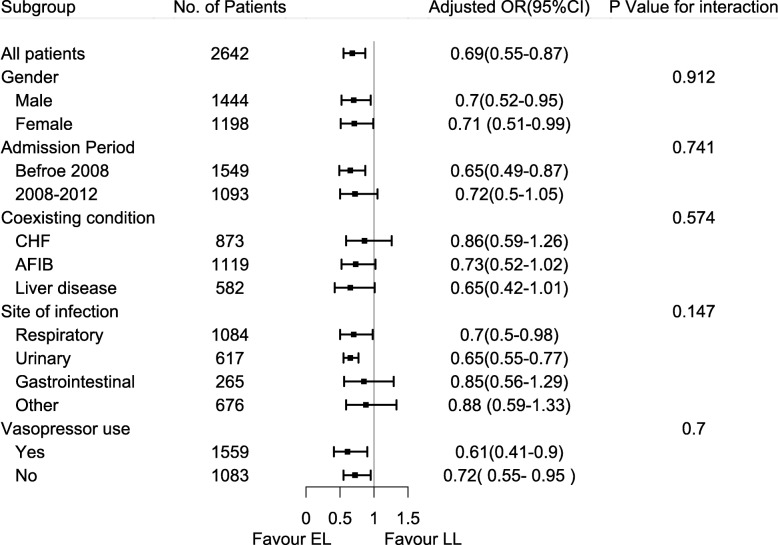
Subgroup analyses of the association between early lactate measurement and 28-day mortality in the original cohort

In the subgroup analyses, regardless of gender or vasopressor use, the associations between early lactate measurement and 28-day mortality were statistically significant, and no significant interaction was detected. Patients admitted before 2008 appeared to have a stronger association than patients admitted during 2008–2012, and no interaction was observed. Other results of the subgroup analyses are presented in Fig. 2.

### Therapeutic interventions and outcomes after PSM

We investigated potential therapeutic interventions that may account for the beneficial effect of early lactate measurement. When administered, patients in the EL group had a shorter time to initial vasopressor administration (2.6 (0.6–5.5) vs. 4.2 (1.0–8.6); *p* < 0.001) and a shorter time to initial antibiotic treatment (1.6 (0.5–4.4) vs. 2.2 (0.8–5.7); *p* = 0.014). There was no significant difference between the two groups in the onset of IVF treatment, but patients in the EL group received more IVF within the first 6 h (4.7 (1.4–9.1) vs. 3.4 (1.0–6.7); *p* < 0.001) and 24 h (11.1 (4.3–21.2) vs. 9.5 (2.9–17.8); *p* = 0.005).

The 28-day mortality rate was lower in the EL group than in the LL group (22.2% vs. 27.5%; *p* = 0.026). The EL group had a shorter duration of vasopressor use (26.6 (24.8–27.4) vs. 23.7 (21.6–24.3); *p* = 0.018). The duration of mechanical ventilation, AKI stage, ICU stay duration, and hospital stay duration did not significantly differ between the two groups. Table 3 shows the detailed results.

**Table 3:** Therapeutic interventions and clinical outcomes after PSM

	EL group	LL group	*p* value
Therapeutic interventions			
Time to initial vasopressor (hours)	2.6 (0.6–5.5)	4.2 (1.0–8.6)	< 0.001
Time to initial antibiotics (hours)	1.6 (0.5–4.4)	2.2 (0.8–5.7)	0.014
Time to initial IVF (hours)	1.8 (0.7–4.7)	1.8 (0.5–5.5)	0.92
Volume of IVF within 6 h (L)	4.7 (1.4–9.1)	3.4 (1.0–6.7)	< 0.001
Volume of IVF within 24 h (L)	11.1 (4.3–21.2)	9.5 (2.9–17.8)	0.005
Clinical outcome			
28-day mortality (%)	22.2%	27.5%	0.026
Ventilation-free days in 28 days	24.1 (17.4–27.2)	23.8 (18.4–27.3)	0.566
Vasopressor-free days in 28 days	26.6 (24.8–27.4)	23.7 (21.6–24.3)	0.018
AKI stage, *n* (%)			0.654
0	52/701 (7.4)	60/701 (8.5)	
1	177/701 (25.2)	168/701 (24)	
2	290/701 (41.3)	277/701 (39.5)	
3	182/701 (25.8)	196/701 (28.0)	
ICU duration (days)	7.2 (3.8–15.1)	7.3 (4–14.1)	0.75
Hospital duration (days)	15 (9–25.2)	16.2 (9.5–26.2)	0.116

*IVF* intravenous fluid, *AKI* acute kidney injury, *ICU* intensive care unit

### Causal mediation analysis

**Fig. 3:**
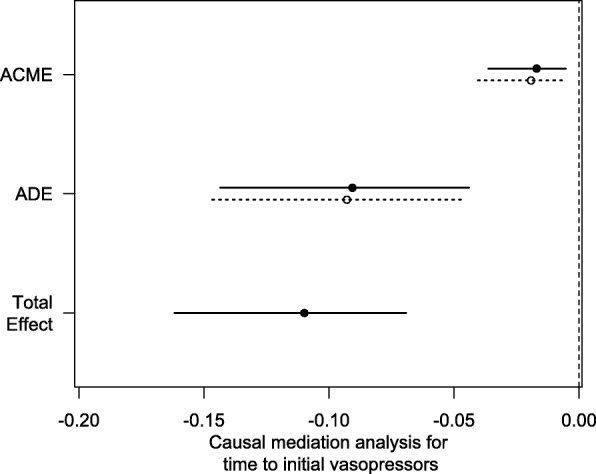
Causal mediation analysis for time to initial vasopressor administration. The solid line represents the early lactate measurement, and the dashed line represents the late lactate measurement

We then used CMA to explore the direct and indirect effects of early lactate measurement on 28-day mortality. The indirect effect was significant only when the time to initial vasopressor administration was used as a mediator variable. The total effect was − 0.11 (95% CI − 0.05 approximately to − 0.174; *p* < 0.001), the ACME was − 0.018 (95% CI − 0.005 approximately to − 0.036; *p* < 0.001), the ADE was − 0.092 (95% CI − 0.03 approximately to − 0.158; *p* < 0.001), and the proportion of the effect mediated was 16.5% (95% CI 4.3%–47%; *p* < 0.001) (Fig. 3). Additionally, an insignificant indirect effect was detected when the time to initial IVF (ACME − 0.003; 95% CI 0.01 approximately to − 0.02; *p* = 0.64) and time to initial antibiotic therapy (ACME − 0.007; 95% CI 0 approximately to − 0.017; *p* = 0.12) were used as mediators (Additional file 1: Figure S3). We concluded that the beneficial effect of early lactate measurement on 28-day mortality is partly mediated through the early administration of vasopressors.

### Delays in lactate measurement and the primary outcome

**Fig. 4:**
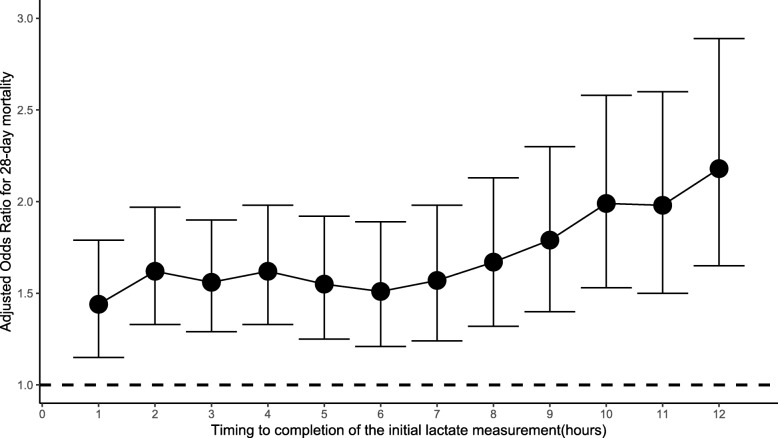
Relationship between the time to complete the initial lactate measurement and 28-day mortality. The odds ratios and 95% confidence intervals (error bars) for each time point were calculated after multivariate adjustment for age, gender, weight, admission type, admission period, severity scores, use of mechanical ventilation, use of RRT, administration of vasopressors, comorbidities, site of infection, MAP, and initial lactate level

**Fig. 5:**
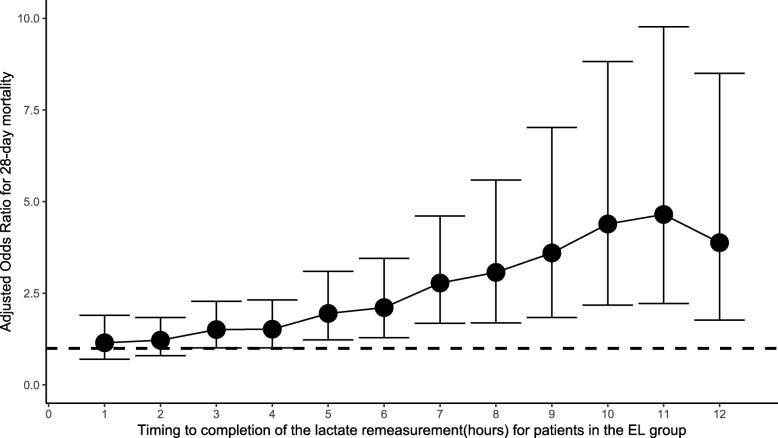
Relationship between the time to complete the lactate remeasurement and 28-day mortality for patients in the EL group. The odds ratios and 95% confidence intervals (error bars) for each time point were calculated after multivariate adjustment for age, gender, weight, admission type, admission period, severity scores, use of mechanical ventilation, use of RRT, administration of vasopressors, comorbidities, site of infection, MAP, and initial lactate level

Multivariate logistic regression analysis showed that a delay in initial lactate measurement was an important determinant of 28-day mortality. The odds ratio (OR) of mortality was 1.04 per hour delay (95% CI 1.02–1.05; *p* < 0.001) (Additional file 1: Table S7). The relationship between the time to complete the initial lactate measurement and 28-day mortality is shown in Fig. 4. In the EL group, 635 patients had more than 1 lactate measurement made (Additional file 1: Table S8). Each hour delay in remeasuring lactate was associated with an increase in 28-day mortality (OR 1.09; 95% CI 1.04–1.15; *p* < 0.001) (Additional file 1: Table S9). Furthermore, there was a consistent increase in the risk of mortality in patients in whom lactate was remeasured more than 3 h later (OR 1.51; 95% CI 1.01–2.27; *p* = 0.042). Figure 5 shows the relationship between the time to complete the lactate remeasurement and 28-day mortality.

## Discussion

Our study revealed that an initial lactate level measurement made within 1 h after ICU admission was associated with lower risk-adjusted 28-day mortality rate in septic patients with an initial lactate level > 2.0 mmol/L, because these patients received earlier vasopressor administration after an early lactate measurement. Regarding the time course of lactate measurement, delays in initial lactate measurement and remeasurement in the EL group were associated with increased risk-adjusted 28-day mortality rates. Our study, for the first time, shows the benefit of early lactate measurement and remeasurement in septic patients with an initial lactate level > 2.0 mmol/L.

Lactate measurement is an important component in the SSC bundle, and the most recent SSC bundle recommends that the initial lactate level should be measured within 1 h to guide resuscitation [9]; however, the suggested timing of the initial lactate measurement is still controversial. Limited studies regarding early lactate measurement in septic patients are available. Seymour et al. [18] reported that a delay in the time to complete the 3-h SSC bundle, including serum lactate measurement, was associated with increased mortality in septic patients after emergency department admission, regardless of the initial lactate level. From 5072 patients with severe sepsis or septic shock, Pruinelli et al. concluded that a delay of 20 min in initial lactate level measurement significantly increased the risk of mortality [19]. Consistent with these findings, our study showed that the 738 patients (27.9%) who had lactate measurements completed within 1 h (EL group) had a lower 28-day mortality rate than did patients who had lactate measurements completed more than 1 h after ICU admission (LL group) after adjustment for confounders, and the relationship was robust after PSM. The present study shows timing evidence that an early lactate measurement is beneficial in sepsis management for septic patients with an initial lactate level > 2.0 mmol/L; specifically, there is a benefit of an initial measurement within 1 h after ICU admission.

Measuring the lactate level itself does not affect clinical outcomes unless it is tied to therapeutic interventions. Several studies have suggested that a timely lactate measurement is associated with more rapid intervention delivery in septic patients. Han et al. [10] demonstrated that early lactate measurement was associated with early antibiotic therapy and IVF in septic patients with initial intermediate or elevated lactate levels, the latter of whom have increased hospital mortality when treatment is delayed [20]. Unfortunately, which therapeutic interventions contribute to the beneficial effect of early lactate measurement on mortality has not been clearly determined. We tested several interventions to interpret the benefit of early lactate measurement and found that the EL group had shorter times to initial vasopressor administration and antibiotic therapy, and although there were no significant differences in the onset of IVF treatment, patients in the EL group received more IVF within 6 h and 24 h. We then used CMA and found that the beneficial effect of early lactate measurement on 28-day mortality in septic patients was partly due to the early administration of vasopressors.

The mediating effect of the time to initial vasopressor administration has been shown by multiple studies. A retrospective cohort study concluded that septic shock patients could benefit from the early administration of norepinephrine [21]. Further randomized trials demonstrated that early norepinephrine administration was strongly associated with more shock control at 6 h, although there was no significant effect on 28-day mortality [22]. Even though we found no pronounced mediation effect of the time to initial IVF and the time to initial antibiotic therapy, these data should not be employed as evidence in sepsis management. There are several explanations for our result. First, it is not the time to antibiotic therapy but the time to appropriate antibiotic therapy that determines the outcome of sepsis [23], and it is difficult for us to test the appropriateness of the antibiotics administered using data from the MIMIC database. Second, the initiation of IVF therapy on the one hand increases the stroke volume, while on the other hand, it contributes to organ edema and dysfunction and increases the duration of organ support [24, 25]. More research on the mediation of therapeutic interventions is needed to assess the effect of early initial lactate measurement on mortality in septic patients.

Our results confirmed the benefit of early lactate measurement, concurrently suggesting that factors that could impact the outcomes of septic patients were complex, such as the type of vasopressor use, appropriate use of antibiotics, choice and volume of resuscitation fluids, corticosteroid use, sedation, and analgesia [26]. Hence, the lactate level should not be seen as the only index to guide resuscitation; but rather, clinicians should make an assessment of the general state of each septic patient and make a comprehensive plan of treatment.

Additionally, multivariate logistic regression analysis in our study further demonstrated that the timing of lactate measurement was an important determinant of 28-day mortality. This association was consistent with the results of the current limited studies. In a retrospective study, the time to the measurement of serum lactate was associated with in-hospital mortality in septic patients (OR 1.04; 95% CI 1.02–1.06; *p* < 0.001). Similar findings were found in Han et al.’s study [10], in which septic patients with initial lactate levels > 2.0 mmol/L had a 2% increased probability of mortality. The OR reported is dissimilar possibly because of the heterogeneity of participants, different primary endpoints, and different adjusted covariates.

To the best of our knowledge, there is no direct information for determining the optimal time for lactate remeasurement in septic patients. The latest SSC bundle recommends [9] that the remeasurement of lactate should be conducted within 2–4 h if the initial lactate level is elevated (> 2.0 mmol/L). However, this recommendation is based on one study by Jasen et al. [6], in which patients with lactate levels > 3.0 mmol/L in the lactate group were treated with the objective of decreasing their lactate levels by 20% or more per 2 h in the initial 8 h of their ICU stays; these patients had a reduced hospital mortality compared to the control group. In our study, we found that remeasuring the lactate level 3 h after the first measurement could affect the outcome in septic patients with lactate levels > 2.0 mmol/L. Our findings provide new evidence supporting the need for clinicians to take earlier action for both the initial lactate measurement and the remeasurement in the management of sepsis.

Our study has several limitations. First, the definition of sepsis was consistent with the third sepsis definition (documented or suspected infection coupled with an acute change in total SOFA score ≥ 2 points), but the diagnoses of infection were unclear [27]. Hence, we tried to include septic patients with positive blood culture results in the sensitivity analysis. Second, data from the MIMIC databases encompass more than 10 years, and the recommendations of the severe sepsis guidelines were changed during the study period, which is a confounding factor affecting the current clinical application of our results. Therefore, we included the admission period (before 2008 and 2008–2012) in our model, and the results were adjusted for the admission period. Third, there are multiple unmeasured confounders [28] that could impact our findings, such as different treatment affecting the lactate level after initial measurement, immunosuppression status, and the interventions before lactate measurement. Fourth, because of the retrospective nature of this cohort study, it is difficult for us to investigate the association between the timing of lactate remeasurement and mortality in all participants; we assessed only the EL group. Fifth, the causes of high lactate levels could be multifactorial in addition to hypoxemia, such as liver disease and drugs, including epinephrine, metformin, and linezolid. Whether the elevated lactate was caused by hypoxemia, drug use, or other diseases was hardly illuminated in the MIMIC-III database. Finally, the causal relationship between early lactate measurements and 28-day mortality was not explored clearly, and the therapeutic interventions after initial lactate measurement were complex, multiple, and often depended on the decision of the clinicians on duty. We examined the mediation effects of the time to initial vasopressor, antibiotic, and IVF administration separately. These effects need to be explored in future studies.

## Conclusion

In conclusion, early lactate measurement (within 1 h after ICU admission) in septic patients with elevated serum lactate levels (> 2.0 mmol/L) is associated with a lower risk-adjusted 28-day mortality. The decrease in time to the administration of vasopressors may have mediated this effect. Repeating the measurement within 3 h after the initial measurement is appropriate for patients whose lactate was measured within 1 h of admission.

## Supplementary information

**Additional file 1: Table S1.** Missing number (%) for included variables in dataset. **Table S2.** Additional demographic data between the early lactate group and the late lactate groups. **Table S3.** Univariate models and full multivariate models assessing the impact of the early lactate measurement and other important factors on 28-day mortality in the original cohort. **Table S4.** Demographic data and comparisons between the early lactate group and late lactate group after matching. **Table S5.** Details of the double robust model on 28-day mortality in the PSM cohorts. **Table S6.** Sensitivity analysis for patients with positive blood culture. **Table S7.** Full multivariate models assessing the impact of timing of the initial lactate level measurements and other important factors on 28-day mortality in the original cohort. **Table S8.** Univariate models and full multivariate models assessing the impact of the timing of lactate remeasurements and other important factors in the early lactate group on 28-day mortality. **Table S9.** Time course of lactate remeasurements for septic patients in the early lactate group. **Figure S1.** Standard mean differences of covariates between the early lactate group and the late lactate group for the original cohort and the matched cohort. **Figure S2.** *p* values measuring the significant differences in covariates for the original cohort and the matched cohort.

## Data Availability

The datasets presented in the current study are available in the MIMIC-III database (https://archive.physionet.org/works/MIMICIIIClinicalDatabase/files/).
