# Supplementary material for: Early lactate measurement is associated with better outcomes in septic patients with an elevated serum lactate level
Source: Crit Care. 2019 Nov 11;23:351. doi: 10.1186/s13054-019-2625-0 (PMC6849274; doi:10.1186/s13054-019-2625-0)
Supplement: Supplementary file 1 — Additional file 1: Table S1. Missing number (%) for included variables in dataset. Table S2. Additional demographic data between the early lactate group and the late lactate groups. Table S3. Univariate models and full multivariate models assessing the impact of the early lactate measurement and other important factors on 28-day mortality in the original cohort. Table S4. Demographic data and comparisons between the early lactate group and late lactate group after matching. Table S5. Details of the double robust model on 28-day mortality in the PSM cohorts. Table S6. Sensitivity analysis for patients with positive blood culture. Table S7. Full multivariate models assessing the impact of timing of the initial lactate level measurements and other important factors on 28-day mortality in the original cohort. Table S8. Univariate models and full multivariate models assessing the impact of the timing of lactate remeasurements and other important factors in the early lactate group on 28-day mortality. Table S9. Time course of lactate remeasurements for septic patients in the early lactate group. Figure S1. Standard mean differences of covariates between the early lactate group and the late lactate group for the original cohort and the matched cohort. Figure S2. p values measuring the significant differences in covariates for the original cohort and the matched cohort. [file 13054_2019_2625_MOESM1_ESM.docx]

Table S1 Missing number (%) for included variables in dataset

| Variables | Missing, n (%) |
| --- | --- |
| Age | 0 (0) |
| Gender | 0 (0) |
| Weight (kg) | 55 (2.1) |
| Admission type | 0 (0) |
| Admission period | 0 (0) |
| Race | 0 (0) |
| Severity of illness |  |
| SOFA score | 0 (0) |
| qSOFA score | 0 (0) |
| SAPS II score | 0 (0) |
| OASIS score | 0 (0) |
| Elixhauser comorbidity score | 0 (0) |
| Interventions |  |
| RRT use (1^st^ 24 h) | 0 (0) |
| Mechanical ventilation use (1^st^ 24 h) | 0 (0) |
| Vasopressor use (1^st^ 24 h) | 0 (0) |
| Comorbidities |  |
| CHF | 0 (0) |
| AFIB | 0 (0) |
| Chronic renal disease | 0 (0) |
| Liver disease | 0 (0) |
| COPD | 0 (0) |
| Stroke | 0 (0) |
| Malignancy | 0 (0) |
| Septic shock | 0 (0) |
| Site of infection |  |
| Respiratory | 0 (0) |
| Urinary | 0 (0) |
| Gastrointestinal | 0 (0) |
| Other | 0 (0) |
| Positive blood cultures | 0 (0) |
| Vital signs |  |
| MAP (mmHg) | 13 (0.5) |
| Heart rate (bpm) | 13 (0.5) |
| Temperature (℃) | 118 (4.5) |
| Respiratory rate(bpm) | 13 (0.5) |
| Initial lactate(mmol/L) | 0 (0) |
| Time to initial vasopressor (hours) | 0 (0) |
| Time to initial antibiotic (hours) | 489 (18.5) |
| Time to initial IVF (hours) | 14 (0.5) |
| AKI stage | 0 (0) |
| ICU duration (days) | 0 (0) |
| Hospital duration (days) | 0 (0) |
| Use of vasopressors | 0 (0) |
| Volume of IVF within 6 hours (L) | 14 (0.5) |
| Volume of IVF within 24 hours (L) | 13 (0.5) |

SOFA: Sequential Organ Failure Assessment; qSOFA: quick Sequential Organ Failure Assessment; SAPS II: Simplified Acute Physiology Score II; OASIS: Overall Anxiety Severity And Impairment Scale; RRT: renal replacement therapy; CHF: congestive heart failure; AFIB: atrial fibrillation; COPD: chronic obstructive pulmonary disease; MAP: mean arterial pressure; IVF: intravenous fluid, AKI: acute kidney injury; ICU: intensive care unit.

Table S2 Additional demographic data between the early lactate group and the late lactate group.

| Variables | EL Group  (n=738) | LL Group  (n=1904) | P value | SMD | |
| --- | --- | --- | --- | --- | --- |
| ICU type, n (%) | | | <0.001 | | 0.167 |
| MICU | 292/738 (39.6) | 850/1904 (44.6) |  |  | |
| CCU/CSRU | 186/738 (25.2) | 530/1904 (27.8) |  |  | |
| TSICU/SICU | 260/738 (35.2) | 524/1904 (27.5) |  |  | |
| Time interval between hospital admission and ICU admission (hours) | 0 (0-16) | 0 (0-18) | 0.669 | 0.022 | |
| Biochemical markers | | | |  | |
| pH | 7.24 (0.131) | 7.27 (0.113) | <0.001 | 0.274 | |
| pO_2_ (mmHg) | 78 (63-98) | 79 (64-102) | 0.168 | 0.096 | |
| pCO_2_ (mmHg) | 32.1 (6.98) | 32.1 (7.41) | 0.984 | 0.001 | |
| PaO2/FiO2 (mmHg) | 156 (102-225) | 160 (103-240) | 0.120 | 0.113 | |
| WBC (*10^9^/L) | 10.35 (6.2-15.2) | 10.3 (6.6-14.8) | 0.954 | 0.001 | |
| Hemoglobin (g/dL) | 9.36 (2.13) | 9.58 (2.22) | 0.017 | 0.103 | |
| Platelet (*10^9^/L) | 141.5 (85-209) | 148 (92-220) | 0.184 | 0.032 | |
| Albumin (g/dL) | 2.65 (0.695) | 2.77 (0.718) | 0.002 | 0.170 | |
| Proportion of different vasopressors, n (%) | | | | | |
| Norepinephrine | 357/738 (48.4) | 928/1904 (48.7) | 0.900 | 0.007 | |
| Epinephrine | 50/738 (6.8) | 173/1904 (9.1) | 0.066 | 0.086 | |
| Dopamine | 109/738 (14.8) | 323/1904 (17.0) | 0.190 | 0.060 | |
| Dobutamine | 53/738 (7.2) | 122/1904 (6.4) | 0.528 | 0.031 | |
| Phenylephrine | 322/738 (43.6) | 804/1904 (42.2) | 0.541 | 0.028 | |
| Vasopressin | 165/738 (22.3) | 385/1904 (20.1) | 0.246 | 0.052 | |

ICU, intensive care unit; MICU, medical intensive care; CCU, coronary care unit; CSRU, cardiac surgery unit; SICU, surgical intensive care unit; TSICU, trauma surgical intensive care unit. WBC; white blood cell.

Table S3 Univariate models and full multivariate model assessing the impact of the early lactate measurement and other important factors on 28-day mortality in the original cohort.

| Variables | Univariate models | | Full multivariate model | |
| --- | --- | --- | --- | --- |
|  | Odds Ratio  (95% CI) | P value | Odds Ratio  (95% CI) | P value |
| Early lactate measurement^*^ | 0.76 (0.62-0.93) | 0.008 | 0.69 (0.55-0.87) | 0.001 |
| Age | 1.02 (1.01-1.02) | <0.001 | 1.01 (1-1.02) | 0.017 |
| Gender (Male) | 0.97 (0.82-1.16) | 0.757 | 0.98 (0.80-1.21) | 0.880 |
| Weight | 0.99 (0.98-0.99) | <0.001 | 0.99 (0.99-1) | 0.087 |
| Admission type | | | | |
| Surgical elective | Reference |  | Reference |  |
| Emergency | 3.28 (2.21,4.88) | <0.001 | 2.32 (1.47-3.66) | <0.001 |
| Surgical emergency | 2.32 (1.21,4.44) | <0.001 | 1.20 (0.57-2.53) | 0.630 |
| Admission period (2008-2012) | 0.89 (0.75-1.06) | 0.201 | 0.75 (0.61-0.92) | 0.005 |
| SOFA | 1.14 (1.11,1.17) | <0.001 | 1.06 (1.02-1.11) | 0.004 |
| qSOFA | 1.36 (1.18,1.57) | <0.001 | 0.83 (0.69-0.99) | 0.049 |
| SAPS II | 1.05 (1.04,1.05) | <0.001 | 1.02 (1-1.04) | <0.001 |
| OASIS | 1.06 (1.05,1.07) | <0.001 | 1.02 (1-1.03) | 0.008 |
| Elixhauser comorbidity score | 1.07 (1.06,1.08) | <0.001 | 1.03 (1.01-1.06) | 0.002 |
| RRT use (1^st^ 24 h) | 1.76 (1.25,2.48) | <0.001 | 0.98 (0.66-1.47) | 0.935 |
| Mechanical ventilation use (1^st^ 24 h) | 0.8 (0.66,0.97) | <0.001 | 0.63 (0.48-0.84) | 0.001 |
| Vasopressor use(1^st^ 24 h) | 1.23 (1.03,1.47) | 0.022 | 0.52 (0.3-0.88) | 0.014 |
| CHF | 1.17 (0.98,1.41) | 0.081 | 0.91 (0.71-1.18) | 0.499 |
| AFIB | 1.16 (0.97,1.38) | 0.097 | 1.16 (0.94-1.43) | 0.177 |
| Chronic renal disease | 1.46 (1.16,1.84) | 0.001 | 0.99 (0.74-1.32) | 0.942 |
| Liver disease | 2.4 (1.98,2.92) | <0.001 | 1.52 (1.08-2.14) | 0.017 |
| COPD | 0.91 (0.73,1.14) | 0.418 | 0.85 (0.66-1.11) | 0.237 |
| Stroke | 0.73 (0.4,1.32) | 0.285 | 0.90 (0.45-1.77) | 0.753 |
| Malignancy | 2.99 (2.34,3.81) | <0.001 | 2.21 (1.56-3.12) | <0.001 |
| Site of infection, |  |  |  |  |
| Respiratory | Reference |  | Reference |  |
| Urinary | 0.9 (0.66,1.22) | 0.494 | 0.65 (0.46-0.92) | 0.014 |
| Gastrointestinal | 0.86 (0.69,1.08) | 0.203 | 0.70 (0.54-0.91) | 0.008 |
| Other | 0.92 (0.74,1.14) | 0.449 | 0.80 (0.62-1.04) | 0.092 |
| Positive blood culture | 1.12 (0.93,1.34) | 0.243 | 0.99 (0.81-1.22) | 0.948 |
| MAP | 0.97 (0.96,0.98) | <0.001 | 0.99 (0.98-1) | 0.338 |
| Initial lactate level | 1.1 (1.06,1.14) | <0.001 | 1.03 (0.99-1.07) | 0.164 |

SOFA: Sequential Organ Failure Assessment; qSOFA: quick Sequential Organ Failure Assessment; SAPS II: Simplified Acute Physiology Score II; OASIS: Overall Anxiety Severity And Impairment scale; RRT: renal replacement therapy; CHF: congestive heart failure; AFIB: atrial fibrillation; COPD: chronic obstructive pulmonary disease; MAP: mean arterial pressure

^*^Early lactate measurement refers to an initial lactate level measured within 1 h after ICU admission.

Table S4 Demographic data and comparisons between the early lactate group and the late lactate group after matching

| Variables | EL Group  (n=701) | LL Group  (n=701) | P value | SMD |
| --- | --- | --- | --- | --- |
| Age (years) | 68 (53-78) | 68 (53-79) | 0.869 | 0.010 |
| Male, n (%) | 396/701 (56.5) | 416/701 (59.3) | 0.304 | 0.058 |
| Weight (kg) | 79.5 (67-93.4) | 81.2 (68.9-86.8) | 0.069 | 0.043 |
| Admission type, n (%) | | | 0.365 | 0.076 |
| Emergency | 598/701 (85.3) | 615/701 (87.7) |  |  |
| Surgical elective | 81/701 (11.5) | 65/701 (9.3) |  |  |
| Surgical urgency | 22/701 (3.1) | 21/701 (3.0) |  |  |
| Admission period, n (%) | | | 0.999 | 0.003 |
| Before 2008 | 411/701 (58.6) | 410/701 (58.5) |  |  |
| 2008-2012 | 290/701 (41.3) | 291/701 (41.5) |  |  |
| Severity of illness | | | | |
| SOFA score | 7.64 (3.47) | 7.70 (3.68) | 0.788 | 0.014 |
| qSOFA score | 2 (2-2) | 2 (2-2) | 0.792 | 0.032 |
| SAPS II score | 47.4 (14.8) | 47.2 (14.1) | 0.819 | 0.012 |
| OASIS score | 38.4 (8.18) | 38.5 (7.89) | 0.826 | 0.012 |
| Elixhauser comorbidity score | 11.3 (8.49) | 10.9 (8.45) | 0.407 | 0.044 |
| Interventions, n (%) | | | | |
| RRT use (1^st^ 24 h) | 36/701 (5.1) | 33/701 (4.7) | 0.805 | 0.020 |
| Mechanical ventilation use (1^st^ 24 h) | 554/701 (79) | 544/701 (77.6) | 0.560 | 0.035 |
| Vasopressor use(1^st^ 24 h) | 430/701 (61.3) | 444/701 (63.3) | 0.474 | 0.041 |
| Comorbidities, n (%) | | | | |
| CHF | 228/701 (32.5) | 218/701 (31.1) | 0.606 | 0.031 |
| AFIB | 285/701 (40.1) | 311/701 (44.3) | 0.177 | 0.075 |
| Chronic renal disease | 108/701 (15.4) | 110/701 (15.7) | 0.941 | 0.008 |
| Liver disease | 138/701 (19.9) | 153/701 (21.8) | 0.357 | 0.075 |
| COPD | 131/701 (18.9) | 133/701 (19.0) | 0.946 | 0.007 |
| Stroke | 15/701 (2.1) | 18/701 (2.6) | 0.725 | 0.028 |
| Malignancy | 83/701 (11.8) | 71/701 (10.1) | 0.348 | 0.055 |
| Site of infection, n (%) | | | | |
| Respiratory | 320/701 (45.6) | 306/701 (43.6) | 0.485 | 0.040 |
| Urinary  Gastrointestinal | 146/701 (20.8)  76/701 (10.8) | 156/701 (22.2)  78/701 (11.1) | 0.559  0.932 | 0.035  0.009 |
| Other | 159/701 (22.7) | 161/701 (23.0) | 0.949 | 0.007 |
| Positive blood cultures, n (%) | 229/701 (32.7) | 225/701 (32.1) | 0.864 | 0.012 |
| MAP(mmHg) | 76.1 (9.6) | 76.6 (10.3) | 0.390 | 0.046 |
| Initial lactate level (mmol/L) | 3.5 (2.7-4.8) | 3.4 (2.6-4.8) | 0.337 | 0.023 |

SOFA: Sequential Organ Failure Assessment; qSOFA: quick Sequential Organ Failure Assessment; SAPS II: Simplified Acute Physiology Score II; OASIS: Overall Anxiety Severity And Impairment Scale; RRT: renal replacement therapy; CHF: congestive heart failure; AFIB: atrial fibrillation; COPD: chronic obstructive pulmonary disease; MAP: mean arterial pressure.

Table S5 Details of the double robust model on 28-day mortality in the PSM cohorts

| Variables | Odds Ratio(95% CI) | P value |
| --- | --- | --- |
| Early lactate measurement^*^ | 0.70 (0.53-0.93) | 0.013 |
| Age | 1.01 (1-1.02) | 0.018 |
| Gender (Male) | 0.98 (0.73-1.31) | 0.885 |
| Weight | 0.99 (0.98-0.99) | 0.040 |
| Admission type | | |
| Surgical elective | Reference | - |
| Emergency | 2.55 (1.28-5.07) | 0.007 |
| Surgical emergency | 1.43 (0.48-4.32) | 0.517 |
| Admission period (2008-2012) | 0.65 (0.48-0.88) | 0.006 |
| SOFA | 1.04 (0.98-1.12) | 0.181 |
| qSOFA | 0.79 (0.60-1.04) | 0.092 |
| SAPS II | 1.03 (1.01-1.05) | 0.001 |
| OASIS | 1 (0.98-1.03) | 0.734 |
| Elixhauser comorbidity score | 1.03 (0.99-1.06) | 0.101 |
| RRT use (1^st^ 24 h) | 0.76 (0.41-1.42) | 0.394 |
| Mechanical ventilation use (1^st^ 24 h) | 0.86 (0.55-1.33) | 0.488 |
| Vasopressor use(1^st^ 24 h) | 0.37 (0.17-0.81) | 0.013 |
| CHF | 0.85 (0.58-1.23) | 0.396 |
| AFIB | 1.29 (0.95-1.75) | 0.107 |
| Chronic renal disease | 0.97 (0.64-1.47) | 0.886 |
| Liver disease | 1.83 (1.12-3.01) | 0.016 |
| COPD | 0.97 (0.67-1.41) | 0.888 |
| Stroke | 0.82 (0.28-2.42) | 0.726 |
| Malignancy | 1.58 (0.98-2.55) | 0.059 |
| Site of infection, | | |
| Respiratory | Reference | - |
| Urinary | 0.84 (0.53-1.34) | 0.475 |
| Gastrointestinal | 0.70 (0.48-1.02) | 0.061 |
| Other | 0.98 (0.68-1.42) | 0.922 |
| Positive blood culture | 0.85 (0.62-1.15) | 0.283 |
| MAP | 1 (0.99-1.02) | 0.627 |
| Initial lactate level | 1.04 (0.97-1.10) | 0.267 |

SOFA: Sequential Organ Failure Assessment; qSOFA: quick Sequential Organ Failure Assessment; SAPS II: Simplified Acute Physiology Score II; OASIS: Overall Anxiety Severity And Impairment scale; RRT: renal replacement therapy; CHF: congestive heart failure; AFIB: atrial fibrillation; COPD: chronic obstructive pulmonary disease; MAP: mean arterial pressure.

^*^Early lactate measurement refers to an initial lactate level measured within 1 h after ICU admission

Table S6 Sensitivity analysis for patients with positive blood culture

|  | Odds ratio | 95% CI | P value |
| --- | --- | --- | --- |
| Model 1 | 0.63 | 0.42-0.94 | 0.023 |
| Model 2 | 0.54 | 0.35-0.83 | 0.005 |
| Model3 | 0.44 | 0.26-0.72 | 0.001 |

Model1, multivariate logistic regression model; Model 2, propensity score matching model; Model 3, doubly robust model with all covariates; CI: confidence interval.

Table S7 Full multivariate model assessing the impact of timing of the initial lactate level measurements and other important factors on 28-day mortality in the original cohort.

| Variables | Odds Ratio (95% CI) | P value |
| --- | --- | --- |
| Timing of the initial lactate measurements | 1.04 (1.02-1.05) | <0.001 |
| Age | 1.01 (1-1.02) | 0.007 |
| Gender(Male) | 0.95 (0.77-1.16) | 0.603 |
| Weight | 1 (1-1) | 0.143 |
| Admission type | | |
| Surgical elective | Reference |  |
| Emergency | 2,32 (1.47-3.67) | <0.001 |
| Surgical emergency | 1.25 (0.6-2.62) | 0.547 |
| Admission period (2008-2012) | 0.79 (0.64-0.98) | 0.031 |
| SOFA | 1.07 (1.02-1.11) | 0.003 |
| qSOFA | 0.83 (0.69-0.99) | 0.042 |
| SAPS II | 1.02 (1-1.03) | <0.001 |
| OASIS | 1.02 (1-1.04) | 0.011 |
| Elixhauser comorbidity score | 1.03 (1.01-1.06) | 0.001 |
| RRT use (1^st^ 24 h) | 0.95 (0.63-1.42) | 0.805 |
| Mechanical ventilation use (1^st^ 24 h) | 0.73 (0.55-0.97) | 0.03 |
| Vasopressor use(1^st^ 24 h) | 0.85 (0.67-1.09) | 0.203 |
| CHF | 0.89 (0.69-1.16) | 0.393 |
| AFIB | 1.15 (0.93-1.42) | 0.189 |
| Chronic renal disease | 0.93 (0.69-1.24) | 0.617 |
| Liver disease | 1.44 (1.02-2.03) | 0.038 |
| COPD | 0.84 (0.65-1.09) | 0.192 |
| Stroke | 0.83 (0.42-1.65) | 0.597 |
| Malignancy | 2.08 (1.47-2.94) | <0.001 |
| Site of infection, | | |
| Respiratory | Reference | - |
| Urinary | 0.67 (0.47-0.94) | 0.02 |
| Gastrointestinal | 0.71 (0.55-0.93) | 0.012 |
| Other | 0.83 (0.64-1.07) | 0.157 |
| Positive blood culture | 0.98 (0.80-1.22) | 0.888 |
| MAP | 0.99 (0.98-1) | 0.313 |
| Initial lactate level | 1.03 (0.98-1.07) | 0.206 |

SOFA: Sequential Organ Failure Assessment; qSOFA: quick Sequential Organ Failure Assessment; SAPS II: Simplified Acute Physiology Score II; OASIS: Overall Anxiety Severity And Impairment scale; RRT: renal replacement therapy; CHF: congestive heart failure; AFIB: atrial fibrillation; COPD: chronic obstructive pulmonary disease; MAP: mean arterial pressure.

Table S8 Univariate models and full multivariate model assessing the impact of the timing of lactate remeasurements and other important factors in the early lactate group on 28-day mortality

| Variables | Univariate models | | | Full multivariate model | |
| --- | --- | --- | --- | --- | --- |
|  | Odds Ratio  (95% CI) | p value | | Odds Ratio  (95% CI) | p value |
| Timing of lactate remeasurements | 1.08 (1.03-1.13) | 0.012 | 1.09 (1.04-1.15) | | <0.001 |
| Age | 1 (1-1) | 0.317 | 1 (1-1) | | 0.507 |
| Gender(Male) | 1.15 (0.79-1.68) | 0.445 | 1.08 (0.71-1.67) | | 0.71 |
| Weight | 1 (0.99-1.01) | 0.114 | 1 (1-1.01) | | 0.275 |
| Admission type | | | | | |
| Surgical elective | Reference |  | Reference | |  |
| Emergency | 0.57 (0.33-0.99) | 0.05 | 0.37 (0.19-0.74) | | 0.005 |
| Surgical emergency | 0.68 (0.2-2.36) | 0.545 | 0.51 (0.13-1.91) | | 0.317 |
| Admission period (2008-2012) | 1.17 (0.81-1.7) | 0.451 | 1.12 (0.74-1.7) | | 0.596 |
| SOFA | 1.07 (1.02-1.13) | 0.012 | 1.09 (1-1.19) | | 0.038 |
| qSOFA | 0.95 (0.7-1.27) | 0.423 | 0.76 (0.52-1.11) | | 0.157 |
| SAPS II | 1 (1-1) | 0.621 | 0.99 (0.97-1.02) | | 0.616 |
| OASIS | 1.01 (0.99-1.03) | 0.573 | 1.03 (0.99-1.06) | | 0.184 |
| Elixhauser comorbidity score | 1.01 (0.99-1.03) | 0.576 | 0.97 (0.93-1.02) | | 0.209 |
| RRT use (1^st^ 24 h) | 1.39 (0.67-2.87) | 0.402 | 1.1 (0.46-2.65) | | 0.828 |
| Mechanical ventilation use (1^st^ 24 h) | 0.98 (0.6-1.62) | 0.671 | 0.56 (0.29-1.09) | | 0.088 |
| Vasopressor use(1^st^ 24 h) | 1.14 (0.77-1.68) | 0.665 | 1.89 (0.3-11.89) | | 0.499 |
| CHF | 1.14 (0.77-1.68) | 0.469 | 1.28 (0.74-2.24) | | 0.381 |
| AFIB | 1.35 (0.93-1.96) | 0.143 | 1.41 (0.9-2.2) | | 0.129 |
| Chronic renal disease | 0.82 (0.48-1.39) | 0.366 | 0.74 (0.39-1.4) | | 0.354 |
| Liver disease | 1.35 (0.87-2.08) | 0.319 | 1.38 (0.66-2.9) | | 0.391 |
| COPD | 0.88 (0.54-1.43) | 0.649 | 0.96 (0.55-1.66) | | 0.87 |
| Stroke | 4.65 (1.59-13.63) | 0.006 | 6.49 (1.94-21.72) | | 0.003 |
| Malignancy | 1.09 (0.62-1.89) | 0.909 | 1.27 (0.63-2.55) | | 0.498 |
| Site of infection, | | | | | |
| Respiratory | Reference |  | Reference | |  |
| Urinary | 0.92 (0.5-1.7) | 0.92 | 0.84 (0.44-1.62) | | 0.611 |
| Gastrointestinal | 1.09 (0.68-1.75) | 0.713 | 1.05 (0.62-1.78) | | 0.845 |
| Other | 0.79 (0.48-1.3) | 0.295 | 0.73 (0.42-1.27) | | 0.261 |
| Positive blood culture | 1.43 (0.97-2.09) | 0.084 | 1.44 (0.95-2.19) | | 0.085 |
| MAP | 1 (0.98-1) | 0.863 | 1 (0.98-1.03) | | 0.749 |
| Initial lactate level | 1.04 (0.97-1.1) | 0.393 | 1.03 (0.95-1.1) | | 0.507 |

SOFA: Sequential Organ Failure Assessment; qSOFA: quick Sequential Organ Failure Assessment; SAPS II: Simplified Acute Physiology Score II; OASIS: Overall Anxiety Severity And Impairment Scale; RRT: renal replacement therapy; CHF: congestive heart failure; AFIB: atrial fibrillation; COPD: chronic obstructive pulmonary disease; MAP: mean arterial pressure.

Table S9 Time course of lactate remeasurements for septic patients in the early lactate group

| Time of lactate remeasurements | No. of patients n (%) | Lactate value (mmol/L) |
| --- | --- | --- |
| 0-1 hour | 126/635(19.8) | 3.7(2.8-5.4) |
| 1-2 hours | 150/635(23.6) | 3.8(2.7-5.8) |
| 2-3 hours | 78/635(12.3) | 3.2(2.2-5.1) |
| 3-4 hours | 63/635(9.9) | 2.9(1.9-4.7) |
| 4-5 hours | 54/635(8.5) | 2.9(2.2-4.2) |
| 5-6 hours | 26/635(4.1) | 3.3(2.0-4.7) |
| 6-7 hours | 29/635(4.6) | 3.0(1.9-4.6) |
| 7-8 hours | 25/635(3.9) | 2.4(1.9-3.7) |
| 8-9 hours | 24/635(3.8) | 2.8(2.0-4.3) |
| 9-10 hours | 16/635(2.5) | 4.4(2.0-7.3) |
| 10-11 hours | 14/635(2.2) | 2.4(1.9-4.1) |
| 11-12 hours | 15/635(2.3) | 3.1(1.7-4.8) |


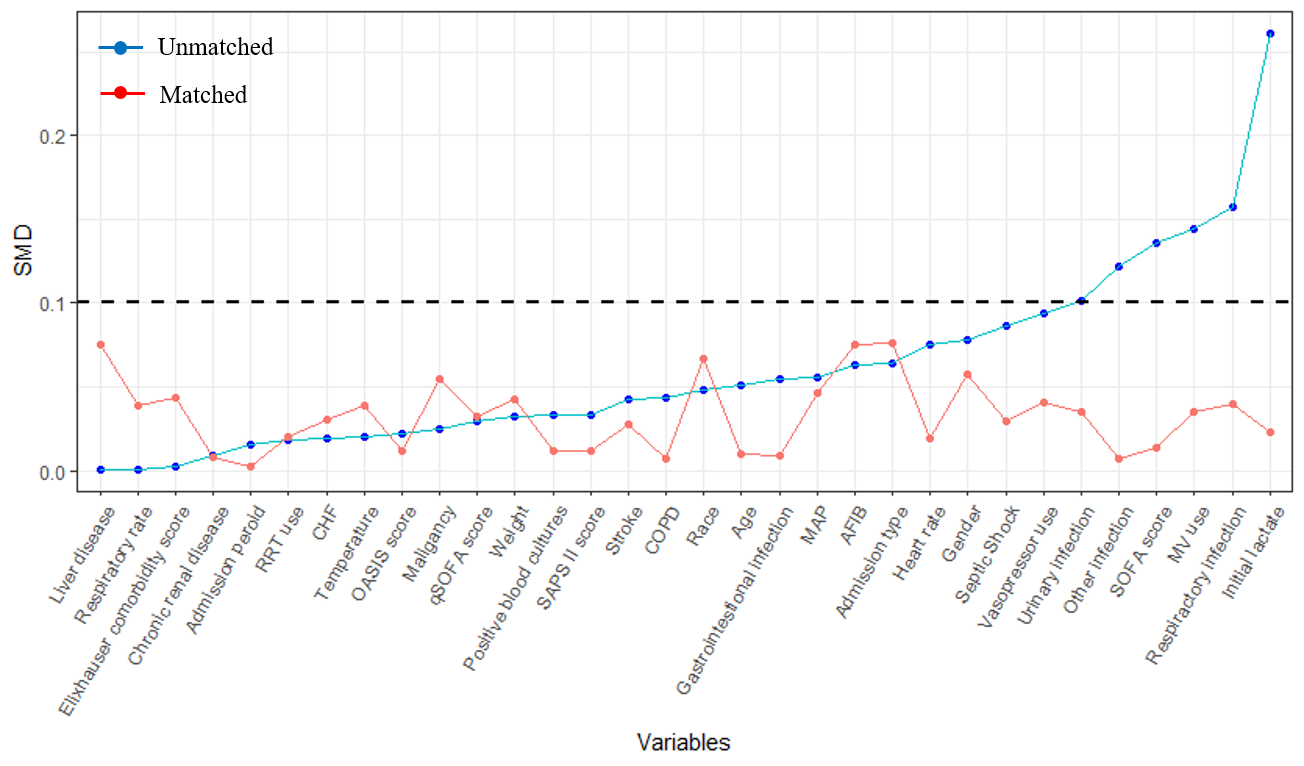


Figure S1 Standard mean differences in covariates between the early lactate group and the late lactate group for the original cohort and the matched cohort.


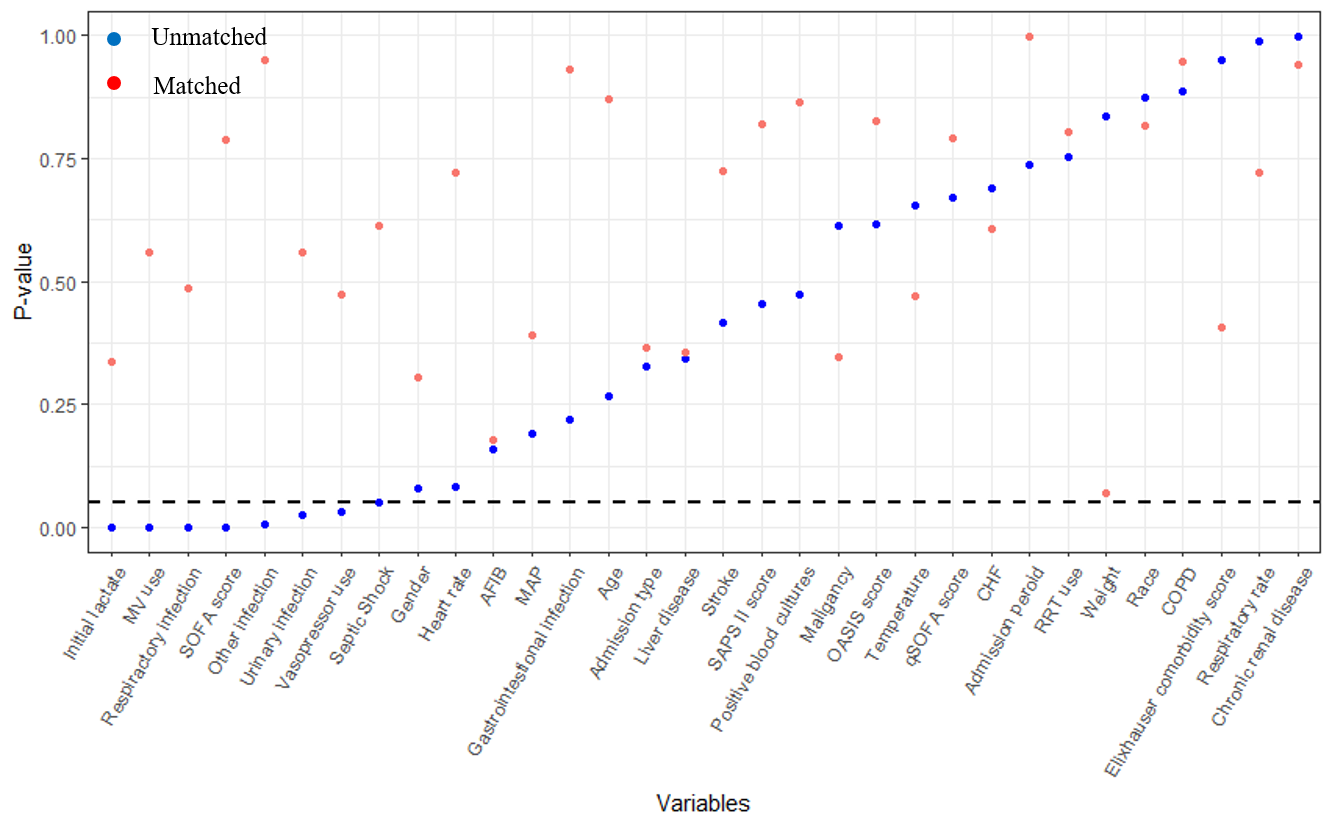


Figure S2 P values measuring the significant differences in covariates for the original cohort and the matched cohort.


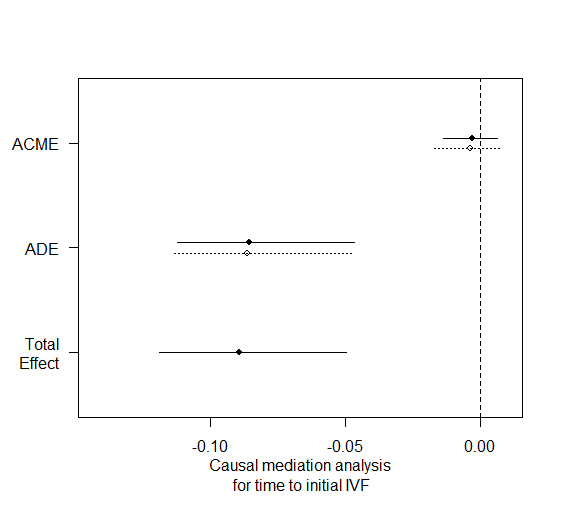

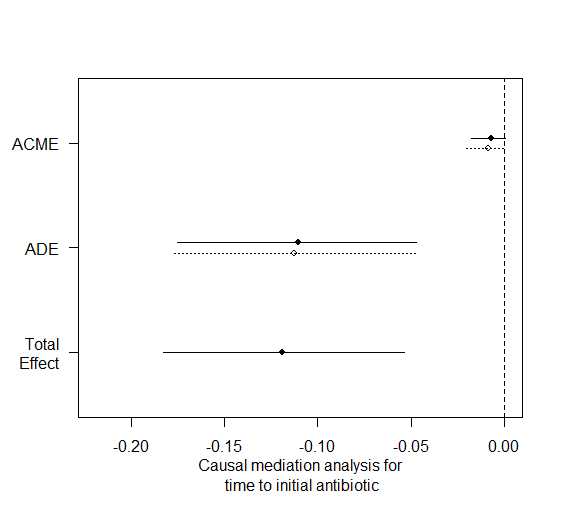


Figure S3 Causal mediation analysis for time to initial IVF and antibiotic. The solid line represents the early lactate measurement, and the dashed line represents the late lactate measurement.
